# Trends and impact of antimicrobial resistance on older inpatients with urinary tract infections (UTIs): A national retrospective observational study

**DOI:** 10.1371/journal.pone.0223409

**Published:** 2019-10-03

**Authors:** Hoa Q. Nguyen, Nga T. Q. Nguyen, Carmel M. Hughes, Ciaran O’Neill

**Affiliations:** 1 School of Pharmacy, Queen's University Belfast, Belfast, United Kingdom; 2 Centre for Public Health, School of Medicine, Dentistry and Biomedical Sciences, Queen's University Belfast, Belfast, United Kingdom; Medical University Graz, AUSTRIA

## Abstract

Urinary tract infections (UTIs) are one of the most common infections in older people and are associated with increased morbidity and mortality. UTIs are also associated with increased risk of antimicrobial resistance (AR). This study examined changes in AR among older inpatients with a primary diagnosis of UTIs in the United States over an 8-year period and the impact of AR on clinical outcomes and hospital costs. Data were obtained from the longitudinal hospital HCUP-NIS database from 2009 to 2016 for inpatient episodes that involved those aged 65+ years. The ICD-9 and ICD-10 codes were used to identify episodes with a primary diagnosis of UTIs, comorbidities, AR status and age-adjusted Deyo-Charlson comorbidity index (ACCI) for the patient concerned. Weighted multivariable regression was used to examine the impact of AR on all-cause inpatient mortality, discharge destination, length of stay and hospital expenditures, adjusted for socio-demographic and clinical covariates. The proportion of admissions with AR increased, from 3.64% in 2009 to 6.88% in 2016 (p<0.001), with distinct patterns for different types of resistance. The likelihood of AR was higher in admissions with high ACCI scores and admissions to hospitals in urban areas. Admissions with AR were more likely to be discharged to healthcare facilities (e.g. care homes) compared to routine discharge (OR 1.81; 95%CI, 1.75–1.86), had increased length of stay (1.12 days; 95%CI, 1.06–1.18) and hospital costs (1259 USD; 95%CI, 1178–1340). Resistance due to MRSA was specifically associated with increased hospital mortality (OR 1.33; 95%CI, 1.15–1.53). Our findings suggest that the prevalence of AR has increased among older inpatients with UTIs in the USA. The study highlights the impact of AR among older inpatients with a primary diagnosis of UTIs on clinical outcomes and hospital costs. These relationships and their implications for the care homes to which patients are frequently discharged warrant further research.

## Introduction

Antimicrobial resistance (AR) is recognized as a global threat to health and healthcare systems. AR could lead to negative impacts on patient outcomes such as increased length of hospital stay, functional decline, increased healthcare expenditure and all-cause mortality [1]. In the United States of America (USA), it is calculated that approximately two million people suffer from infections which are resistant to antimicrobials each year and at least 23,000 die of these conditions [2]. By 2050, it is estimated that AR will trigger 10 million deaths worldwide every year [3]. In addition, AR has a massive economic impact; in the USA, for example it is estimated to cost approximately $2.2 billion annually and adds $1,383 for every patient treated [4]. The challenge presented by AR has also increased over time with the appearance of new resistant strains [2,5].

Older people are more susceptible to AR due to physiological changes and comorbidity [6]. This population has potentially higher risks of developing AR-related illness due to higher exposure to infection from hospital and institutional settings [7,8]. Long-term care facilities for older people may also increase exposure to AR because they have been recognized as reservoirs of resistant bacteria and have high rates of antimicrobial prescribing [9–11]. In the USA, the percentage of older people admitted to hospital with resistant infections increased by 48.8% from 1997 to 2006 [12]. The population of older persons is expected to increase globally to 1.4 billion by 2030 [13]; therefore, AR may become a key consideration in the management of older people’s care in the future.

Urinary tract infections (UTIs) are one of the most common infections in older people and are associated with increased morbidity and mortality [7,8]. Difficulty in diagnosis of UTIs among older people may lead to complications such as bacteremia if untreated [14] or *Clostridium difficile* infection if treated inappropriately [15]. High rates of AR have also been recorded amongst patients with UTIs [16–18]. This may contribute to poor outcomes of treating older patients with UTIs.

There is a paucity of evidence in respect of the impact of AR on the treatment and outcomes of older patients with UTIs in an inpatient setting. This study aimed to examine changes in AR over time and its impact on clinical outcomes and hospital costs among older people hospitalized with UTIs in the USA.

## Materials and methods

### Data source

We conducted a retrospective observational study using the National (Nationwide) Inpatient Sample—Healthcare Cost and Utilization Project (HCUP-NIS), Agency for Healthcare Research and Quality (AHRQ). The HCUP-NIS is the largest available all-payer inpatient healthcare administrative dataset in the USA corresponding to an approximately 20 percent stratified sample of all discharges from community hospitals but excluding rehabilitation and long-term acute care hospitals [19]. Each record includes patient demographic details, diagnoses, procedures and other information associated with a single hospital admission. Data in relation to medication use, including antimicrobials, and microbiological results were not available; however, administrative datasets without such data have the potential to explore the impact of AR [12,20]. Due to the de-identified and publicly-available nature of HCUP-NIS data, this study was considered exempt by the School Research Ethics Committee/Institutional Review Board at Queen’s University Belfast.

### Case definition

We obtained HCUP-NIS data from 2009 to 2016. The study cohort was restricted to episodes of care that involved those aged 65 years and over, the age commonly used to define older persons in the USA [21]. The International Classification of Diseases 9th Revision and 10th Revision (ICD-9 and ICD-10) primary procedure and diagnostic codes were used to identify admissions with a primary diagnosis of UTIs. Following coding practice in the USA, ICD-9 codes were used for data before the fourth quarter of 2015, and ICD-10 codes were used for data from the fourth quarter of 2015 until the end of 2016. Cases were identified as admissions with a primary diagnosis of UTIs based on the definition of AHRQ Prevention Quality Indicator 12 for Urinary Tract Infections [22]. This definition comprises urinary tract infection, acute cystitis, cystitis, acute pyelonephritis, renal/perirenal abscess, pyeloureteritis cystica, pyelonephritis or pyonephrosis not specified as acute or chronic, and infection of kidney; it excludes cases with any codes for kidney/urinary tract disorder or transferred from another health care facility.

Infections due to Methicillin-resistant *Staphylococcus aureus* (MRSA) have been associated with high rates of mortality and prolonged hospital stay [23], and MRSA is the only resistant organism for which specific codes have been created by the ICD system since October 2008 [24]. Therefore, we decided to report resistance due to MRSA separately from other types of AR. Episodes with all types of AR, including beta-lactam resistance (BR), resistance due to MRSA, multidrug resistance (MR), quinolone resistance (QR), were identified using ICD codes (See S1 Appendix). We were also particularly interested in BR, resistance due to MRSA, MR, and QR as these types of resistance can develop in patients with UTIs [18] and have a major impact on patient outcomes [3,5]. AR was defined as having any ICD-9 V09.XX codes or diagnosis codes for MRSA (038.12, 041.12, 482.42), and equivalent ICD-10 codes. The ICD-9 codes were also used to define BR (V09.0, V09.1), QR (V09.5X), and MR (V09.81, V09.91), along with equivalent ICD-10 codes. An age-adjusted Deyo-Charlson Comorbidity Index (ACCI) was established using ICD codes to identify any of the following comorbidities for each admission: congestive heart failure, chronic pulmonary disease, cerebrovascular disease, diabetes mellitus with or without chronic complications, dementia, myocardial infarction, rheumatic disease, peripheral vascular disease, mild, moderate or severe liver disease, peptic ulcer disease, renal disease, hemiplegia or paraplegia, and HIV/AIDS. We used the Deyo-Charlson Comorbidity Index as this provided details on ICD-9 codes to define comorbidities, and applied age-adjusted version of Charlson Comorbidity Index as older patient data were used in this study [25,26]. Episodes with comorbidities were then weighted using the Deyo-Charlson algorithm and ACCI score calculated [25,26].

### Study variables

HCUP-NIS provided a range of socio-demographic variables comprising insurance type (Medicare/Medicaid/Private/Others), ethnicity, sex (male/female), age at admission, median household income for patients’ ZIP code and location (broadly in terms of rurality). In order to control for hospital characteristics, we used the unique HCUP hospital number to link the core data to the hospital data. These variables comprised a hospital's ownership/control category (government/private owned), location (rural, urban), and its teaching status (non-teaching, teaching). Clinical variables included the comorbidity index ACCI (as calculated above), mode of presentation (elective/non-elective) and total number of procedures performed in the hospital. We also identified cases with sepsis, a severe complication of UTIs, and *C*. *difficile* infection, a common complication of long-term use of broad-spectrum antibiotics potentially due to UTIs treatment, using ICD codes (see S1 Appendix for more details). These variables were chosen because they were either reported to be associated with mortality [14,27] or found to be clinically relevant in the literature [28–31].

### Study outcomes

We first described the characteristics of admissions with UTIs, proportion of admissions with AR in general and with specific types of resistance (BR, resistance due to MRSA, MR, and QR). The four main outcomes associated with AR, comprising all-cause inpatient mortality, discharge destination, length of stay in the hospital, and hospital incurred costs, were measured to estimate the impact of AR [1]. Discharge destination included routine discharge (i.e. discharge to home, self-care, and court/law enforcement), and discharge to healthcare facilities (including long-term care facilities or care homes, short-term hospitals, home healthcare and other rehabilitation centers), excluding those who died at the hospital. The hospital-specific all-payer inpatient ‘cost-to-charge’ ratio tool provided by AHRQ-HCUP was used to convert discharges to hospital costs. Hospital costs were then adjusted for inflation using the personal consumption expenditure health component price index based on its ability to capture information on expenditures by all payers [32,33]. This is based on the assumption that average all-payer reimbursements or expenditures could be a proxy for underlying resource costs, a common practice used in literature [32].

### Statistical analyses

Sample characteristics were described using median and interquartile range (IQR) for continuous variables with skewed distribution, mean and standard deviation (SD) for other continuous variables and proportions for categorical variables. In order to generate national estimates using HCUP-NIS data that span multiple years, all analyses were weighted by trend weight for data years prior to 2012 and by the discharge-level weight for data from 2012 and later as recommended by the AHRQ [19] unless specified otherwise. This practice reflects the major change in sampling method of HCUP-NIS in 2012 (changing from a 20% stratified sample of hospitals to a 20% national patient-level sample, with non-representative sampling across hospitals) [19].

#### Trends in antimicrobial resistance

The incidence of UTIs as a primary diagnosis and proportion of admissions with AR, BR, resistance due to MRSA, MR, or QR were described on a monthly basis and weighted to provide national estimates. Multivariable logistic regression was used to examine factors affecting the likelihood of having AR, BR, MR, and QR. Explanatory variables used in these analyses comprised socio-demographic variables (insurance type, ethnicity, sex, age at admission, median household income, location, and year), and clinical variables (comorbidity score ACCI, hospital characteristics, mode of presentation) as suggested by the literature [28–31].

#### Impact of antimicrobial resistance

We estimated the impact of AR by examining the four outcomes: all-cause inpatient mortality, discharge to another healthcare facility, length of stay in the hospital, and hospital incurred costs. We examined factors associated with the likelihood of inpatient mortality and discharge to a healthcare facility among those admitted with a primary diagnosis of UTIs using weighted multivariable logistic regression. Models were adjusted for socio-demographic variables (insurance type, ethnicity, gender, age at admission, median household income, location), and clinical variables (comorbidity score ACCI, hospital teaching status, hospital location, hospital ownership, mode of presentation, number of procedures performed, *C*. *difficile* infection and sepsis status). Year was added as a predictor to control for variability over time in all regression models.

Similarly, based on the nature of count data and evidence of over-dispersion of length of stay in our data, we fitted weighted negative binomial regression models to examine the impact of AR, BR, resistance due to MRSA, MR, and QR on length of stay. Generalized linear models (GLM) were used to accommodate the continuous, positive and skewed nature of hospital cost data in the cost analysis. Akaike information criterion (AIC) and Bayesian information criterion (BIC) were used to assess the fit of the GLM model [34]. The link function and distribution family were jointly chosen using AIC and BIC while running a series of GLM models. Marginal effect analyses were used to estimate the incremental hospital costs attributable to AR, BR, resistance due to MRSA, MR, and QR.

### Sensitivity analyses

The UTI definition of AHRQ Prevention Quality Indicator 12 for Urinary Tract Infections includes acute pyelonephritis, renal/perirenal abscess, pyeloureteritis cystica, pyelonephritis or pyonephrosis not specified as acute or chronic, and infection of kidney [22]. Such conditions have been recognized to result in serious complications or treatment failure [35]. Therefore, we conducted a set of sensitivity analyses with a refined sample without ICD codes for acute pyelonephritis, renal/perirenal abscess, pyeloureteritis cystica, pyelonephritis or pyonephrosis not specified as acute or chronic, and infection of kidney.

All analyses and data manipulations were performed using STATA version 15 (StataCorp LLC, College Station, TX).

## Results

### Characteristics of the cohort

Over the 8-year period, a total of 546,305 eligible admissions with a primary diagnosis of UTIs were included in the study. Characteristics of episodes with primary UTIs are presented in Table 1. Most of the cohort was white female, with more White American and fewer females in the AR group and resistance due to MRSA group. Medicare was the predominant type of insurance, accounting for approximately 92% in all groups, while more people in the non-AR group had private insurance. Admissions with AR were more likely to be in the highest income group, those who were slightly younger, had more comorbidities, increased length of stay and higher hospital costs. Among subgroups of AR, resistance due to MRSA group had the highest cost while those with MR were the youngest and most likely to have private insurance.

**Table 1:** Characteristics of the pooled cohort from 2009–2016.

	Non-AR (N = 521947)		AR (N = 24358)		BR (N = 2708)		MRSA (N = 9712)		MR (N = 6630)		QR (N = 1434)	
Age, mean (SD)^a^	80.8	7.8	80.6***	7.9	80.5	7.8	81.0*	7.8	79.9***	8.0	81.3*	7.8
Female, N (%)^b^	357524	68.5%	14402***	59.1%	1859	68.7%	4303***	44.3%	4,489	67.7%	995	69.4%
Insurance, N (%)^b^												
Medicare	481479	92.3%	22599***	92.8%	2509***	92.7%	9032***	93.0%	6,089***	91.8%	1338	93.3%
Medicaid	7406	1.4%	324***	1.3%	61***	2.3%	100***	1.0%	106***	1.6%	15	1.1%
Private	26964	5.2%	1095***	4.5%	113***	4.2%	440***	4.5%	321***	4.8%	65	4.5%
Income, N (%)^b^												
Lowest income quartile	152,142	29.2%	6847***	28.1%	682***	25.2%	2828	29.1%	1926***	29.1%	328***	22.9%
Second lowest income quartile	132711	25.4%	5994***	24.6%	645***	23.8%	2464	25.4%	1582***	23.9%	364***	25.4%
Second highest income quartile	124312	23.8%	5812***	23.9%	674***	24.9%	2312	23.8%	1532***	23.1%	367***	25.6%
Highest income quartile	112782	21.6%	5705***	23.4%	707***	26.1%	2108	21.7%	1590***	24.0%	375***	26.2%
Ethnicity, N (%)^b^												
White Americans	407207	78.0%	19189*	78.8%	2036***	75.2%	7894***	81.3%	5017***	75.7%	1151	80.3%
Black Americans	54036	10.4%	2418*	9.9%	243***	9.0%	965***	9.9%	672***	10.1%	131	9.1%
Hispanic Americans	38511	7.4%	1696*	7.0%	271***	10.0%	506***	5.2%	600***	9.1%	87	6.1%
Asian Americans	9047	1.7%	431*	1.8%	74***	2.7%	150***	1.5%	128***	1.9%	23	1.6%
Native Americans	2350	0.5%	120*	0.5%	15***	0.6%	41***	0.4%	35***	0.5%	10	0.7%
Others	10796	2.1%	504*	2.1%	69***	2.6%	156***	1.6%	178***	2.7%	32	2.2%
Hospital type, N (%)^b^												
Private hospital	460266	88.2%	21341**	87.6%	2360	87.2%	8529	87.8%	5763**	86.9%	1233**	86.0%
Hospital in urban area	437181	83.8%	20429	83.9%	2286	84.4%	8052*	82.9%	5566	84.0%	1185	82.6%
Teaching hospital	224081	42.9%	10478	43.0%	1232**	45.5%	3895***	40.1%	2991***	45.1%	590	41.1%
Discharge destination, N (%)												
Routine discharge ^b^	186,650	35.8%	5556***	22.8%	750***	27.7%	1727***	17.8%	1799***	27.1%	430***	30.0%
Discharge to healthcare facility ^b^	327654	62.8%	18376***	75.4%	1929***	71.2%	7736***	79.7%	4761***	71.8%	990***	69.0%
ACCI, mean (SD)^a^	5.7	2.2	6.1***	2.3	6.1***	2.2	6.1***	2.3	5.9***	2.2	5.9**	2.2
Length of stay, median (IQR)^c^, day	3	2–5	5***	3–7	4***	3–6	5***	4–8	5***	3–7	4***	3–5
Hospital costs, median (IQR)^c^, 2016 USD	5641	3901–8413	7383***	4924–11414	7021***	4833–10478	8038***	5403–12548	6757***	4457–10415	6194***	4286–8947

Note:

* p<0.05,

** p<0.01,

*** p<0.001.

All tests used non-AR group as the base category.

^a^ Independent sample t-test,

^b^ Chi-square test

^c^, Man-Whitney U-test,

IQR: interquartile range, SD: Standard error, AR: antimicrobial resistance, BR: beta-lactam resistance, MRSA: resistance due to MRSA, MR: multidrug resistance, QR: quinolone resistance. AR group includes those with BR, MRSA, MR, and QR.

### Trends in antimicrobial resistance

**Fig 1:**
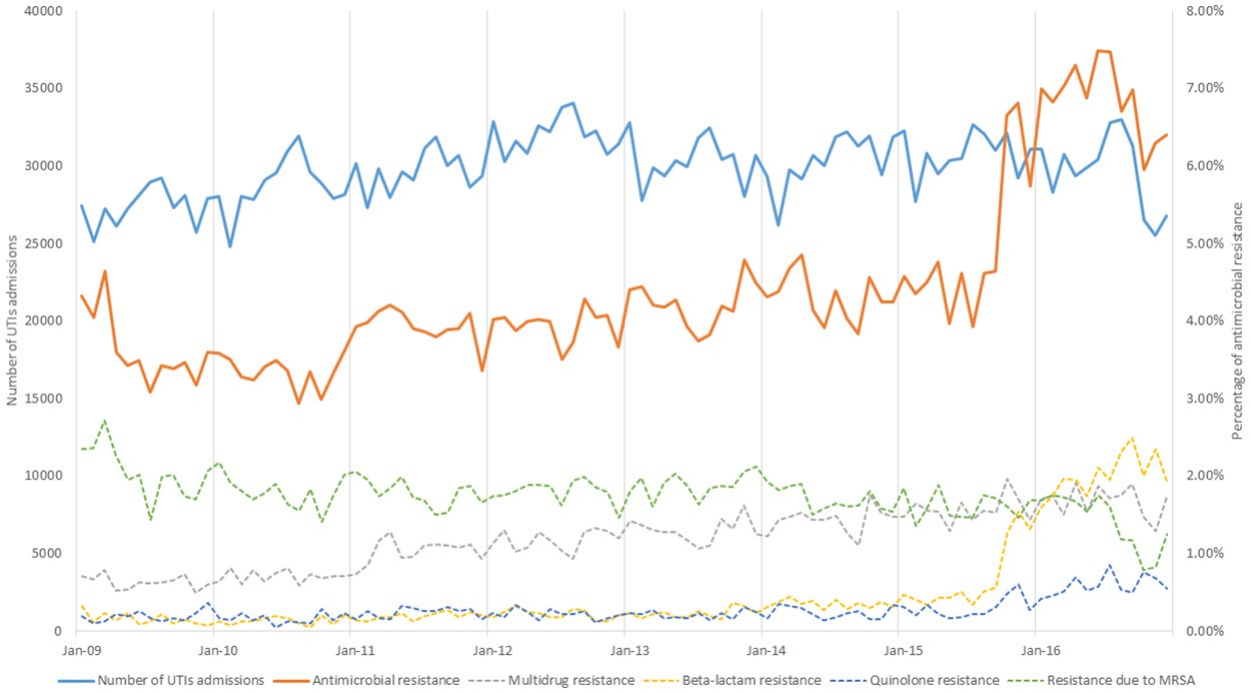
Trends in primary UTIs admission and antimicrobial resistance from 2009–2016.

Fig 1 describes the total number of admissions with a primary diagnosis of UTIs and the proportion of cases with any type of AR by month. All data were weighted by HCUP-NIS weights to reflect the national estimates over the 8-year period. In the pooled data, while UTIs admission rate fluctuated over this period with a seasonal peak in the summer months, there was also an upward trend in AR with a gradual upward trend in MR and QR, a sharp increase in BR in 2015 and a downward trend in resistance due to MRSA. The proportion of AR among inpatient episodes those aged 65+ with a primary diagnosis of UTIs increased from 3.64% in 2008 to 6.88% in 2016 (p<0.001).

### Factors associated with the likelihood of AR, BR, MR and QR

**Fig 2:**
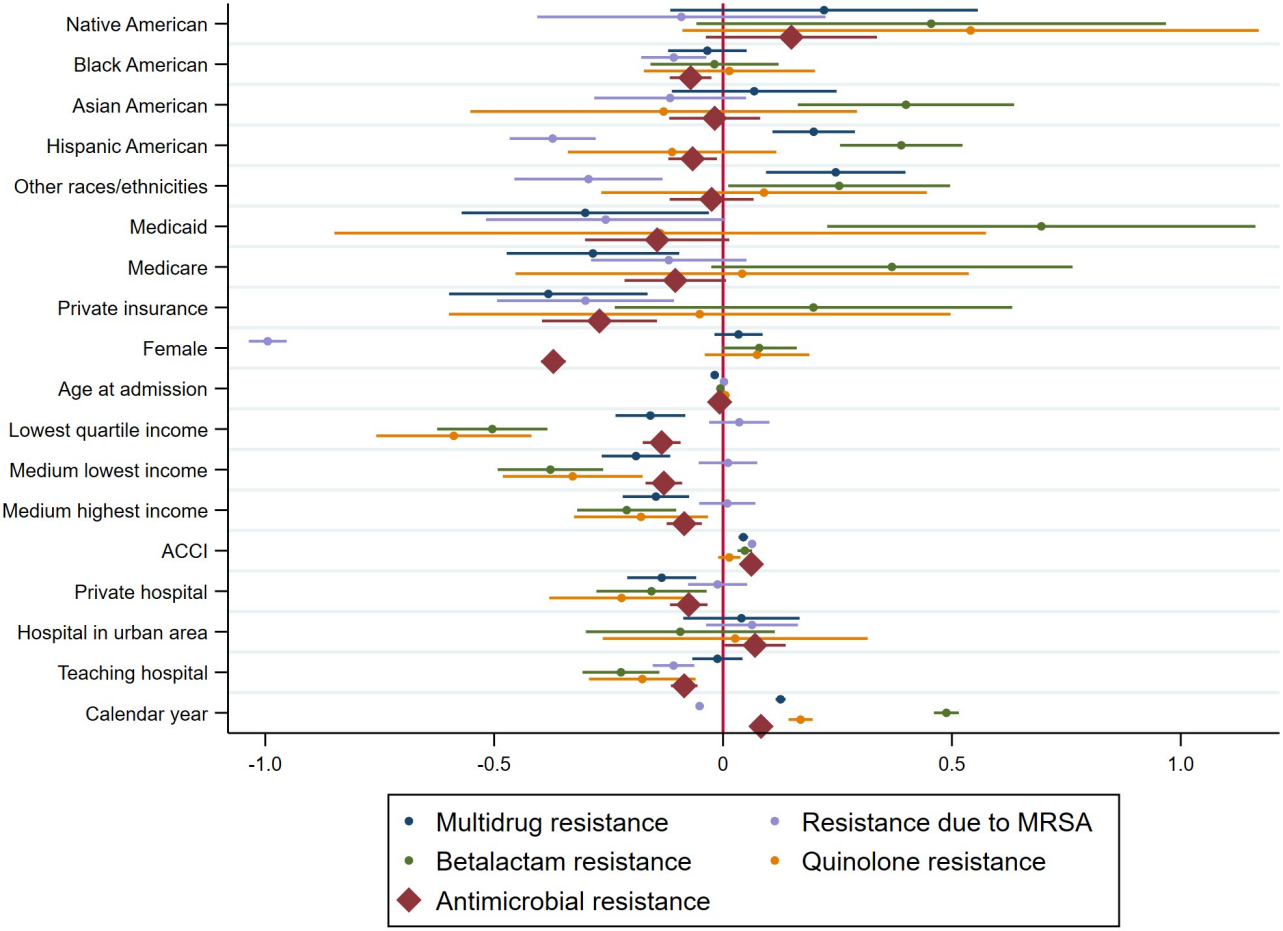
Factors associated with AR, BR, resistance due to MRSA, MR and QR.

As shown in Fig 2, factors associated with higher likelihood of having AR included higher ACCI score (OR 1.06, 95%CI, 1.06–1.07) and admissions to hospitals in urban areas (OR 1.07, 95%CI, 1.00–1.15). On the other hand, factors associated with lower likelihood of having AR included being Black American (OR 0.93, 95%CI, 0.89–0.98), being Hispanic (OR 0.94, 95%CI, 0.89–0.99), having private insurance (OR 0.76, 95%CI, 0.67–0.87), being female (OR 0.69, 95%CI, 0.67–0.71), having low income, and admission to a private (OR 0.93, 95%CI, 0.89–0.97) or teaching hospital (OR 0.91, 95%CI, 0.89–0.95) and increased age (OR 0.99, 95%CI, 0.99–0.99). A comparable pattern was observed among those with BR or MR that factors were associated with increased likelihood of having BR or MR included being Asians, Hispanics or other races, higher income, and higher ACCI. Notably, a decreased likelihood of having resistance due to MRSA was found among those who were Black, Hispanic Americans and other ethnicities, female, had lower ACCI, and private insurance. S2 Appendix provides further details on the likelihood of having different types of AR.

### Impact of antimicrobial resistance

#### All-cause inpatient mortality

Over the 8-year period, the all-cause inpatient mortality rate was 1.42% for UTI admissions with associated AR, compared to 1.09% in the non-AR group (p < 0.001). In the multivariable logistic model, we found non-significant difference in the odds of death between the two groups during the observed inpatient episode (see Table 2). However, when examining specific types of AR, (BR, resistance due to MRSA, MR, and QR), we found a higher risk of in-patient death among those with resistance due to MRSA (OR 1.33, 95%CI, 1.15–1.53), but lower risk among those with BR (OR 0.48, 95%CI, 0.28–0.80), and MR (OR 0.67, 95%CI, 0.51–0.89). Other factors that were predictive of mortality were having *C*. *difficile* infection, having sepsis, being Black American, older age and higher ACCI. Moreover, having any types of insurance (Medicaid/Medicare/private insurance), being admitted to private hospital or hospital in an urban area reduced risk of inpatient mortality. Risk of inpatient mortality also fell over time.

**Table 2:** Impact of antimicrobial resistance.

	AR	BR	MRSA	MR	QR
**Unadjusted models**					
All-cause inpatient mortality^a^, OR (95%CI)	1.31 (1.17–1.45)	0.52 (0.32–0.85)	2.11 (1.85–2.40)	0.69 (0.53–0.91)	0.87 (0.51–1.47)
Discharge to healthcare facilities^a^, OR (95%CI)	1.81 (1.76–1.86)	1.40 (1.29–1.52)	2.29 (2.18–2.4)	1.47 (1.40–1.55)	1.20 (1.08–1.34)
Length of stay^b^ Days (95%CI)	1.77 (1.70 to 1.84)	0.94 (0.64 to 1.24)	2.41 (2.28 to 2.54)	1.25 (1.14 to 1.35)	-0.11 (-0.27 to 0.04)
Hospital costs^c^ 2016 USD (95%CI)	2730 (2596 to 2864)	1654 (1349 to 1960)	3957 (3708 to 4207)	1562 (1349 to 1775)	34 (-250 to 318)
**Adjusted models**^Φ^					
All-cause inpatient mortality^a^, OR (95%CI)	1.03 (0.92–1.15)	0.48 (0.28–0.80)	1.33 (1.15–1.53)	0.67 (0.51–0.89)	0.86 (0.49–1.50)
Discharge to healthcare facilities^a^, OR (95%CI)	1.81 (1.75–1.86)	1.39 (1.28–1.52)	2.21 (2.10–2.33)	1.57 (1.49–1.66)	1.20 (1.07–1.35)
Length of stay^b^ Days (95%CI)	1.12 (1.06 to 1.18)	0.74 (0.44 to 1.03)	1.36 (1.27 to 1.46)	0.83 (0.74 to 0.92)	-0.05 (-0.19 to 0.10)
Hospital costs^c^ 2016 USD (95%CI)	1259 (1178 to 1340)	910 (679 to 1141)	1653 (1521 to 1784)	646 (511 to 781)	122 (-111 to 356)

Note:

^a^ Logistic models;

^b^ Negative binomial regression model,

^c^ Generalized linear models,

^Φ^Models were adjusted for a range of socio-demographic and clinical covariates as stated in the Method section.

All models were weighted for HCUP weights to generate national estimates, the reference cases were those without antimicrobial resistance. OR: odds ratio, 95%CI: 95% confidence interval, AR: antimicrobial resistance, BR: beta-lactam resistance, MRSA: resistance due to MRSA, MR: multidrug resistance, QR: quinolone resistance. AR group includes those with BR, resistance due to MRSA, MR, and QR.

#### Discharge destination

Among survivors, 22.8% of patients with AR were routinely discharged, while 75.4% were transferred to other healthcare facilities (including long-term care facilities, short-term hospitals, home healthcare or other rehabilitation centers), compared to 35.8% and 62.8% in the non-AR group, respectively. Multivariable regression showed that admissions with any types of AR were more likely to be discharged to a healthcare facility than those without resistance. Among different types of AR, admissions with resistance due to MRSA showed the highest odds of being discharged to a health facility, which was over two times higher than those without resistance (OR 2. 21, 95%CI, 2. 10–2. 33) (see Table 2).

#### Length of stay in the hospital

Compared to those without AR, the unadjusted additional length of stay for admissions with AR was 1. 77 days (p<0.001). After being adjusted for other covariates, admissions with AR were 1. 12 days (95%CI, 1. 06–1. 18) longer in hospital compared to non-AR. The predicted length of stay of those with BR, resistance due to MRSA, and MR were 0.74, 1.36, 0.83 days longer than those without AR, respectively (see Table 2). There was no significant difference in length of stay between admissions with QR and those without AR.

#### Hospital incurred costs

Hospital discharges were converted to costs using cost-to-charge ratio provided by the HCUP and were inflated to the 2016 value. The unadjusted costs associated with hospitalization with AR were 2730 USD (95%CI, 2596–2864) higher than non-AR group (p<0.001). After estimating a series of GLM models, the AIC and BIC statistics supported the use of log link function and distribution family gamma. In the multivariable regression, admissions with AR, on average, consumed 1259 USD (95%:1178–1340) more than those without AR, though distinct patterns were observed in different types of AR (see Table 2).

### Sensitivity analyses

Results of the sensitivity analyses on a refined sample without upper UTIs diagnosis confirmed our findings (see S3 Appendix). The general patterns of factors associated with increased/decreased likelihood of having AR, BR, resistance due to MRSA, MR, and QR remained comparable to the main analysis. Regarding impact of antimicrobial resistance, having AR was associated with increased likelihood of being discharged to a healthcare facility (OR 1.74, 95%CI, 1.69–1.80), increased hospital stays by 1.11 days (95%CI, 1.05–1.18), and increased hospital costs by 1236 USD (95%CI, 1152–1320). Resistance due to MRSA remained the costliest group with 1.35 days longer hospital stay, 2.12 times more likely to have healthcare discharge, and 1.29 times more likely to die in hospital.

## Discussion

While the prevalence of AR has been shown to increase over time [5], especially in hospital admissions with infections [12], to our knowledge, this is the first study to explore trends in the impact of AR on older inpatients with UTIs. Our findings suggest that AR among older inpatients with UTIs increased over time in the USA from 2009 to 2016. Socio-demographic factors associated with the likelihood of resistance were gender, age, median household income, type of insurance, ethnicity, ACCI score, and type of admitting hospital. Patients with AR were associated with an increased likelihood of discharge to other healthcare facilities, increased length of stay, hospital costs and all-cause inpatient mortality, although distinct patterns were evident between specific types of AR.

Within our study, the number of admissions with UTIs did not significantly change during the 8-year period of observation; however, there was a distinct seasonal pattern. Summer peaks among episodes with UTIs has been reported in a previous study [36]. Episodes with recorded AR did, however, increase substantially with distinct patterns for BR, QR, and MR except for resistance due to MRSA. The slight decrease over time in percentage of resistance due to MRSA has been also reported in literature [37,38]. However, this trend in our study may only be representative of inpatients aged from 65 with UTIs, and ICD codes for MRSA have been reported to have less sensitivity in detecting cases with MRSA [39]. The sharp rise of AR and BR from October 2015 may be partly due to introduction of the ICD-10 codes for infection with extended spectrum beta-lactamase resistance, which had not been recorded separately in the ICD- 9 codes. Although antimicrobial stewardship programs have been widely promoted since 2007 [40], AR prevalence seems to have risen continuously regardless, which suggests better and more intensive approaches should be implemented.

Risk factors associated with the likelihood of AR among inpatients with UTIs in our study are consistent with findings in previous studies. High ACCI scores indicated multiple comorbidities which are considered as a risk factor for antimicrobial resistance [31,41]. Male patients with UTIs were more likely to develop resistance compared to female patients [30,42]; this is perhaps unsurprising as older men are likely to develop UTI complications [14]. Additionally, we found that although male admissions were younger (mean age 79.8 ±7.8) compared to female (mean age 81.3±7.8) (p<0.001), they had higher ACCI scores (mean ACCI 5.9±2.4) compared to female (mean ACCI 5.6±2.1) (p<0.001). This could partly explain the lower risk of AR resistance among female in our cohort. Regarding ethnicities, we found that being Black or Hispanic is associated with lower odds of having AR compared to Whites. Compared to White Americans (mean 5.7±2.2), Black American had higher ACCI score (mean 6.0±2.3), and Hispanic Americans had lower ACCI score (mean 5.6±2.2) (p<0.001). While one may suspect higher ACCI among White admissions could contribute to their observed higher likelihood of AR, we suppose that there may be further unobserved factors such as the use of antimicrobials among this subgroup. In fact, White persons have been reported to be prescribed higher amounts of antimicrobials than other ethnicities including Black and Hispanic persons [28] which may help to explain the higher rates of AR among this group. The increased likelihood of BR and MR among Asian and Hispanic persons could be due to the fact that they were carriers of resistance organisms from Asian and Latin American countries where high rates of AR and antibiotic consumption have been reported [43].

We found that increased age was associated with a decreased likelihood of AR, BR and MR; nevertheless, the AR and MR group had higher ACCI and lower age compared to the non-AR group (Table 1). This could suggest that inpatients with AR or MR who had high ACCI may not have lived as long as those without AR. Admissions to hospitals in urban areas were also associated with an increased likelihood of having AR. This was perhaps due to a high density of people and hospitals in urban areas which may accelerate transmission of AR bacteria [44]. We also found that having health insurance (including private insurance) or having lower income was associated with lower risk of AR. A previous study also reported an increased AR-associated infection risk among patients without health insurance [12]. Additionally, although individual data of outpatient healthcare expenditure was not available, populations with high income may be more likely to pay for private healthcare, which has been associated with antimicrobial resistance [29,45], perhaps again related to easier access to antimicrobials. One might argue that people with high income would be likely to have private insurance, thus the AR pattern should be consistent between these groups. However, in HCUP-NIS, income-related data were constructed based on median household income for patients’ ZIP code; a degree of caution is, therefore, warranted in the interpretation of this result given the potential for ecological fallacy.

With regard to the burden of AR, episodes with recorded resistance due to MRSA were associated with an increased likelihood in inpatient mortality and an increase in length of hospital stay, which is consistent with findings of previous studies [46,47]. Interestingly, episodes with BR and MR were associated with lower risks of hospital death. However, we found episodes with BR and MR were 1.39 and 1.57 times more likely to be discharged to healthcare facilities (including long-term care facilities or care homes) compared to those without resistance. It should also be noted that ACCI scores in the BR and MR groups were both significantly higher than those in the non-AR group (Table 1). Although there was no clear reason to explain a reduction in inpatient observed mortality among inpatients with BR or MR, it is possible that patients with BR or MR were severely ill so perhaps the decision was made to discharge them to other long-term care facilities for end-of life care [48]. Higher number of admissions with AR sent to healthcare facilities at discharge may well contribute to the reservoirs of antimicrobial resistance in this setting [10,11] an issue that may be particularly concerning given the risk this presents to others. Our findings were also similar to those of previous studies which reported that AR infection increased length of hospital stay and hospital expenditure [46,47,49]. The addition to hospital costs associated with AR in our study was lower than those reported previously which was presented for specific resistant strains such as MRSA or Extended spectrum beta-lactamase resistance-producing *Escherichia coli* [46,47]. The full cost of infections should ideally include those associated with subsequent care, where we did not factor in the higher likelihood of transfer to another care facility among those with AR. The data available to us does not permit this follow-up however. We note though the recommendations made elsewhere in respect of the better estimation of economic impact [46,50]. Given these recommendations, we adopted a robust approach to utilize a representative sample of the population of interest as well as choosing GLM models to accommodate the distinct nature of costs data while taking into account potential confounding factors. These approaches represented our endeavor to increase the reliability of the estimated costs while noting the limitations imposed by the data.

The current study used a population-based data which represented 20% of national inpatient data in the USA. By pooling data in 8 years, the large number of episodes supported the study power. However, our study had several limitations. Chronological age (e.g. 65 years and over) is commonly used as cut-off to define older populations but its usefulness as an indicator for physiological and functional status of the older population has been disputed [51]. Frailty, a condition of functional decline among older people, has been reported to be associated with adverse inpatient outcomes, including hospital mortality [52,53]. We were unable to identify older inpatients with frailty using ICD codes for this administrative dataset. However, as proportions of frailty increase with age in the US older population [54], we used an age-adjusted version of CCI score as a proxy of health status and frailty of our cohort, and subsequently adjusted all models for this variable. We noted the development of an ICD-10 code for sarcopenia, but as this did not become available until September 2016, we were unable to exploit it in our analyses. We concur, however, that in future releases of HCUP data, the use of this code to distinguish between older persons in terms of frailty may benefit this research area.

Moreover, HCUP-NIS data are collected mainly for administrative purposes; thus, important clinical data such as medication use or microbiological results were not available. The cross-sectional nature of this study also limited our ability to make further suggestions regarding our findings. In addition, primary and secondary diagnoses and antimicrobial resistance were identified using ICD codes which have been reported with potentially missing cases [24]. Nevertheless, we endeavored to exploit available information in the data which could be surrogate measures for clinical data such as complications, types of resistance or discharge information. Medication use data during hospital admission may not be necessary to explore AR as patients may have developed resistance due to transmission or previous antimicrobial use. In order to reduce bias from ICD codes, we applied the definition of UTIs using ICD codes by AHRQ and used all codes that clearly defined resistant infections in ICD-9 and ICD-10 [22,24]. As noted, we cannot observe what happens to patients after discharge and cannot therefore state confidently as to longer term mortality or healthcare cost implications of AR. However, even with these caveats, our findings indicate a considerable impact of this issue on clinical and economic outcomes.

## Conclusions

Our findings suggest that AR is increasing among older inpatients with UTIs. We also found distinct patterns in the relationship between specific types of AR and the likelihood of all-cause inpatient mortality, hospital discharge destination, length of stay, and hospital costs. These relationships and their implications for the care homes to which patients are likely discharged warrant further research.

## Supporting information

S1 Appendix ICD codes used to identify antimicrobial resistance. (DOCX)

S2 Appendix Factors associated with the likelihood of antimicrobial resistance. (DOCX)

S3 Appendix Sensitivity analyses. (DOCX)
