# Supplementary material for: Trends and impact of antimicrobial resistance on older inpatients with urinary tract infections (UTIs): A national retrospective observational study
Source: PLoS One. 2019 Oct 3;14(10):e0223409. doi: 10.1371/journal.pone.0223409 (PMC6776395; doi:10.1371/journal.pone.0223409)
Supplement: S1 Appendix — (DOCX) [file pone.0223409.s001.docx]

**S1 APPENDIX. ICD CODES USED TO IDENTIFY ANTIMICROBIAL RESISTANCE**

**ANTIMICROBIAL RESISTANCE**

| **ICD-9** | **ICD-10** |
| --- | --- |
| V09.XX Infection with drug-resistant microorganisms | Z16.XX Resistance to antimicrobial drugs |
| 38.12 Methicillin resistant *Staphylococcus aureus* septicaemia | A41.02 Sepsis due to Methicillin resistant *Staphylococcus aureus* |
| 41.12 Methicillin resistant *Staphylococcus aureus* in conditions classified elsewhere and of unspecified site | B95.62 Methicillin *resistant Staphylococcus aureus* infection as the cause of diseases classified elsewhere  A49.02 Methicillin resistant *Staphylococcus aureus* infection, unspecified site |
| 482.42 Methicillin resistant pneumonia due to *Staphylococcus aureus* | J15.212 Pneumonia due to Methicillin resistant *Staphylococcus aureus* |

**BETA-LACTAM RESISTANCE**

| **ICD-9** | **ICD-10** |
| --- | --- |
| V09.0 Infection with microorganisms resistant to penicillins  V09.1 Infection with microorganisms resistant to cephalosporins and other beta-lactam antibiotics | Z16.1 Resistance to beta-lactam antibiotics |

**RESISTANCE DUE TO METHICILLIN RESISTANT *STAPHYLOCOCCUS AUREUS***

| **ICD-9** | **ICD-10** |
| --- | --- |
| 38.12 Methicillin resistant *Staphylococcus aureus* septicaemia | A41.02 Sepsis due to Methicillin resistant *Staphylococcus aureus* |
| 41.12 Methicillin resistant *Staphylococcus aureus* in conditions classified elsewhere and of unspecified site | B95.62 Methicillin *resistant Staphylococcus aureus* infection as the cause of diseases classified elsewhere  A49.02 Methicillin resistant *Staphylococcus aureus* infection, unspecified site |
| 482.42 Methicillin resistant pneumonia due to *Staphylococcus aureus* | J15.212 Pneumonia due to Methicillin resistant *Staphylococcus aureus* |

**QUINOLONE RESISTANCE**

| **ICD-9** | **ICD-10** |
| --- | --- |
| V09.5 Infection with microorganisms resistant to quinolones and fluoroquinolones | Z16.23 Resistance to quinolones and fluoroquinolones |

**MULTIDRUG RESISTANCE**

| **ICD-9** | **ICD-10** |
| --- | --- |
| V09.81 Infection with microorganisms with resistance to multiple drugs | Z16.35 Resistance to multiple antimicrobial drugs |
| V09.91 Infection with drug-resistant microorganisms, unspecified, with multiple drug resistance | Z16.24 Resistance to multiple antibiotics |

**SEPSIS DUE TO INFECTIONS**

| **ICD-9** | **ICD-10** |
| --- | --- |
| 790.7 Bacteremia | R78.81 Bacteremia |
| 038 Septicemia | A40 Streptococcal sepsis |
|  | A41 Other sepsis |

**CLOSTRIDIUM DIFFICILE INFECTION**

| **ICD-9** | **ICD-10** |
| --- | --- |
| 008.45 Intestinal infection due to *Clostridium difficile* | A04.71 Enterocolitis due to *Clostridium difficile*, recurrent  A04.72 Enterocolitis due to *Clostridium difficile*, not specified as recurrent |
