# Supplementary material for: Trends and impact of antimicrobial resistance on older inpatients with urinary tract infections (UTIs): A national retrospective observational study
Source: PLoS One. 2019 Oct 3;14(10):e0223409. doi: 10.1371/journal.pone.0223409 (PMC6776395; doi:10.1371/journal.pone.0223409)
Supplement: S2 Appendix — (DOCX) [file pone.0223409.s002.docx]

**S2 APPENDIX. FACTORS ASSOCIATED WITH THE LIKELIHOOD OF ANTIMICROBIAL RESISTANCE**

**Table S1.** Factors associated with the likelihood of having antimicrobial resistance

| Antimicrobial resistance | Odds Ratio | SE | P-value | 95% CI | |
| --- | --- | --- | --- | --- | --- |
|  |  |  |  |  |  |
| Native American | 1.161 | 0.111 | 0.117 | 0.963 | 1.400 |
| Black American | 0.932 | 0.022 | 0.002 | 0.890 | 0.975 |
| Asian American | 0.982 | 0.050 | 0.720 | 0.889 | 1.085 |
| Hispanic American | 0.936 | 0.025 | 0.014 | 0.887 | 0.987 |
| Other ethnicities | 0.975 | 0.046 | 0.595 | 0.890 | 1.069 |
| Medicaid insurance | 0.866 | 0.070 | 0.073 | 0.740 | 1.014 |
| Medicare insurance | 0.901 | 0.051 | 0.065 | 0.806 | 1.007 |
| Private insurance | 0.763 | 0.049 | <0.001 | 0.673 | 0.866 |
| Female | 0.690 | 0.009 | <0.001 | 0.672 | 0.709 |
| Age | 0.993 | 0.001 | <0.001 | 0.991 | 0.994 |
| Lowest income quartile | 0.875 | 0.018 | <0.001 | 0.839 | 0.912 |
| Second lowest income quartile | 0.879 | 0.018 | <0.001 | 0.844 | 0.915 |
| Second highest income quartile | 0.919 | 0.018 | <0.001 | 0.884 | 0.955 |
|  |  |  |  |  |  |
| Location |  |  |  |  |  |
| Fringe counties of metro areas of >=1 million population | 0.954 | 0.018 | 0.012 | 0.920 | 0.990 |
| Counties in metro areas of 250,000-999,999 population | 0.915 | 0.019 | <0.001 | 0.879 | 0.953 |
| Counties in metro areas of 50,000-249,999 population | 0.914 | 0.025 | 0.001 | 0.868 | 0.964 |
| Micropolitan counties | 1.020 | 0.037 | 0.590 | 0.949 | 1.096 |
| Not metropolitan or micropolitan counties | 0.925 | 0.035 | 0.039 | 0.859 | 0.996 |
|  |  |  |  |  |  |
| ACCI | 1.064 | 0.003 | <0.001 | 1.058 | 1.070 |
| Private hospital | 0.928 | 0.019 | <0.001 | 0.891 | 0.967 |
| Hospital in urban area | 1.072 | 0.037 | 0.041 | 1.003 | 1.147 |
| Teaching hospital | 0.919 | 0.014 | <0.001 | 0.892 | 0.946 |
| Elective admission | 1.544 | 0.042 | <0.001 | 1.464 | 1.629 |
| Year | 1.087 | 0.003 | <0.001 | 1.080 | 1.093 |

**Table S2.** Factors associated with the likelihood of having multidrug resistance

| Multidrug resistance | Odds Ratio | SE | P-value | 95%CI | |
| --- | --- | --- | --- | --- | --- |
|  |  |  |  |  |  |
| Native American | 1.247 | 0.214 | 0.198 | 0.891 | 1.745 |
| Black American | 0.966 | 0.042 | 0.434 | 0.887 | 1.053 |
| Asian American | 1.071 | 0.098 | 0.458 | 0.894 | 1.282 |
| Hispanic American | 1.219 | 0.056 | <0.001 | 1.114 | 1.334 |
| Other ethnicities | 1.279 | 0.099 | 0.002 | 1.098 | 1.490 |
| Medicaid insurance | 0.740 | 0.102 | 0.029 | 0.565 | 0.970 |
| Medicare insurance | 0.753 | 0.072 | 0.003 | 0.623 | 0.909 |
| Private insurance | 0.683 | 0.076 | 0.001 | 0.550 | 0.848 |
| Female | 1.035 | 0.028 | 0.206 | 0.981 | 1.090 |
| Age | 0.982 | 0.002 | <0.001 | 0.979 | 0.985 |
| Lowest income quartile | 0.853 | 0.033 | <0.001 | 0.791 | 0.921 |
| Second lowest income quartile | 0.827 | 0.032 | <0.001 | 0.767 | 0.891 |
| Second highest income quartile | 0.864 | 0.032 | <0.001 | 0.803 | 0.929 |
|  |  |  |  |  |  |
| Location |  |  |  |  |  |
| Fringe counties of metro areas of >=1 million population | 0.928 | 0.032 | 0.031 | 0.867 | 0.993 |
| Counties in metro areas of 250,000-999,999 population | 0.784 | 0.031 | <0.001 | 0.726 | 0.847 |
| Counties in metro areas of 50,000-249,999 population | 0.814 | 0.042 | <0.001 | 0.736 | 0.900 |
| Micropolitan counties | 0.971 | 0.066 | 0.669 | 0.850 | 1.110 |
| Not metropolitan or micropolitan counties | 0.739 | 0.053 | <0.001 | 0.641 | 0.851 |
|  |  |  |  |  |  |
| ACCI | 1.045 | 0.006 | <0.001 | 1.034 | 1.056 |
| Private hospital | 0.875 | 0.034 | <0.001 | 0.811 | 0.943 |
| Hospital in urban area | 1.041 | 0.068 | 0.535 | 0.917 | 1.182 |
| Teaching hospital | 0.988 | 0.028 | 0.662 | 0.935 | 1.044 |
| Elective admission | 2.321 | 0.103 | <0.001 | 2.127 | 2.532 |
| Year | 1.134 | 0.006 | <0.001 | 1.122 | 1.146 |

**Table S3.** Factors associated with the likelihood of having resistance due to MRSA

| Multidrug resistance | Odds Ratio | SE | P-value | 95%CI | |
| --- | --- | --- | --- | --- | --- |
|  |  |  |  |  |  |
| Native American | 0.913 | 0.147 | 0.571 | 0.666 | 1.251 |
| Black American | 0.898 | 0.033 | 0.003 | 0.836 | 0.964 |
| Asian American | 0.891 | 0.075 | 0.172 | 0.755 | 1.052 |
| Hispanic American | 0.689 | 0.033 | <0.001 | 0.627 | 0.757 |
| Other ethnicities | 0.745 | 0.062 | <0.001 | 0.634 | 0.876 |
| Medicaid insurance | 0.774 | 0.103 | 0.055 | 0.596 | 1.005 |
| Medicare insurance | 0.888 | 0.077 | 0.171 | 0.750 | 1.053 |
| Private insurance | 0.740 | 0.073 | 0.002 | 0.610 | 0.898 |
| Female | 0.370 | 0.008 | <0.001 | 0.355 | 0.385 |
| Age | 1.002 | 0.001 | 0.147 | 0.999 | 1.005 |
| Lowest income quartile | 1.036 | 0.035 | 0.291 | 0.970 | 1.107 |
| Second lowest income quartile | 1.011 | 0.033 | 0.739 | 0.948 | 1.078 |
| Second highest income quartile | 1.009 | 0.032 | 0.766 | 0.949 | 1.073 |
|  |  |  |  |  |  |
| Location |  |  |  |  |  |
| Fringe counties of metro areas of >=1 million population | 0.993 | 0.030 | 0.807 | 0.936 | 1.053 |
| Counties in metro areas of 250,000-999,999 population | 1.052 | 0.034 | 0.112 | 0.988 | 1.120 |
| Counties in metro areas of 50,000-249,999 population | 1.005 | 0.042 | 0.907 | 0.926 | 1.090 |
| Micropolitan counties | 1.053 | 0.060 | 0.360 | 0.943 | 1.177 |
| Not metropolitan or micropolitan counties | 1.087 | 0.062 | 0.141 | 0.973 | 1.215 |
|  |  |  |  |  |  |
| ACCI | 1.066 | 0.004 | <0.001 | 1.057 | 1.074 |
| Private hospital | 0.988 | 0.033 | 0.717 | 0.926 | 1.054 |
| Hospital in urban area | 1.065 | 0.054 | 0.216 | 0.964 | 1.178 |
| Teaching hospital | 0.898 | 0.021 | <0.001 | 0.858 | 0.939 |
| Elective admission | 1.117 | 0.051 | 0.016 | 1.021 | 1.223 |
| Year | 0.950 | 0.004 | <0.001 | 0.942 | 0.959 |

**Table S4.** Factors associated with the likelihood of Beta-lactam resistance

| Beta-lactam resistance | Odds Ratio | SE | P>z | 95%CI | |
| --- | --- | --- | --- | --- | --- |
|  |  |  |  |  |  |
| Native American | 1.576 | 0.412 | 0.082 | 0.943 | 2.632 |
| Black American | 0.981 | 0.070 | 0.793 | 0.853 | 1.129 |
| Asian American | 1.491 | 0.180 | 0.001 | 1.177 | 1.889 |
| Hispanic American | 1.476 | 0.101 | <0.001 | 1.291 | 1.687 |
| Other ethnicities | 1.289 | 0.159 | 0.040 | 1.011 | 1.642 |
| Medicaid insurance | 2.004 | 0.478 | 0.004 | 1.255 | 3.200 |
| Medicare insurance | 1.446 | 0.291 | 0.067 | 0.975 | 2.146 |
| Private insurance | 1.218 | 0.270 | 0.372 | 0.789 | 1.881 |
| Female | 1.082 | 0.045 | 0.059 | 0.997 | 1.175 |
| Age | 0.994 | 0.003 | 0.026 | 0.989 | 0.999 |
| Lowest income quartile | 0.604 | 0.037 | <0.001 | 0.535 | 0.681 |
| Second lowest income quartile | 0.686 | 0.040 | <0.001 | 0.611 | 0.770 |
| Second highest income quartile | 0.810 | 0.045 | <0.001 | 0.727 | 0.903 |
|  |  |  |  |  |  |
| Location |  |  |  |  |  |
| Fringe counties of metro areas of >=1 million population | 0.877 | 0.048 | 0.016 | 0.788 | 0.976 |
| Counties in metro areas of 250,000-999,999 population | 1.001 | 0.059 | 0.990 | 0.892 | 1.123 |
| Counties in metro areas of 50,000-249,999 population | 0.969 | 0.077 | 0.691 | 0.830 | 1.132 |
| Micropolitan counties | 0.882 | 0.100 | 0.268 | 0.706 | 1.101 |
| Not metropolitan or micropolitan counties | 0.955 | 0.108 | 0.686 | 0.765 | 1.193 |
|  |  |  |  |  |  |
| ACCI | 1.049 | 0.009 | <0.001 | 1.032 | 1.066 |
| Private hospital | 0.855 | 0.053 | 0.011 | 0.758 | 0.965 |
| Hospital in urban area | 0.911 | 0.096 | 0.376 | 0.741 | 1.120 |
| Teaching hospital | 0.800 | 0.034 | <0.001 | 0.736 | 0.870 |
| Elective admission | 1.266 | 0.112 | 0.007 | 1.065 | 1.505 |
| Year | 1.629 | 0.023 | <0.001 | 1.585 | 1.674 |

**Table S5.** Factors associated with the likelihood of Quinolone resistance

| Quinolone resistance | Odds Ratio | SE | P>z | 95%CI | |
| --- | --- | --- | --- | --- | --- |
|  |  |  |  |  |  |
| Native American | 1.717 | 0.552 | 0.092 | 0.915 | 3.223 |
| Black American | 1.014 | 0.097 | 0.884 | 0.841 | 1.222 |
| Asian American | 0.878 | 0.189 | 0.547 | 0.576 | 1.340 |
| Hispanic American | 0.895 | 0.104 | 0.339 | 0.712 | 1.124 |
| Other ethnicities | 1.094 | 0.198 | 0.622 | 0.766 | 1.561 |
| Medicaid insurance | 0.872 | 0.317 | 0.705 | 0.428 | 1.776 |
| Medicare insurance | 1.043 | 0.263 | 0.869 | 0.635 | 1.711 |
| Private insurance | 0.950 | 0.266 | 0.856 | 0.549 | 1.644 |
| Female | 1.077 | 0.063 | 0.202 | 0.961 | 1.208 |
| Age | 1.005 | 0.004 | 0.158 | 0.998 | 1.012 |
| Lowest income quartile | 0.555 | 0.048 | <0.001 | 0.469 | 0.658 |
| Second lowest income quartile | 0.720 | 0.056 | <0.001 | 0.618 | 0.839 |
| Second highest income quartile | 0.836 | 0.062 | 0.017 | 0.722 | 0.968 |
|  |  |  |  |  |  |
| Location |  |  |  |  |  |
| Fringe counties of metro areas of >=1 million population | 0.905 | 0.068 | 0.180 | 0.781 | 1.047 |
| Counties in metro areas of 250,000-999,999 population | 1.035 | 0.085 | 0.677 | 0.881 | 1.214 |
| Counties in metro areas of 50,000-249,999 population | 0.967 | 0.107 | 0.763 | 0.779 | 1.201 |
| Micropolitan counties | 1.328 | 0.203 | 0.065 | 0.983 | 1.793 |
| Not metropolitan or micropolitan counties | 1.050 | 0.175 | 0.768 | 0.758 | 1.456 |
|  |  |  |  |  |  |
| ACCI | 1.014 | 0.013 | 0.269 | 0.989 | 1.039 |
| Private hospital | 0.801 | 0.065 | 0.006 | 0.684 | 0.938 |
| Hospital in urban area | 1.027 | 0.152 | 0.857 | 0.769 | 1.372 |
| Teaching hospital | 0.838 | 0.050 | 0.003 | 0.746 | 0.942 |
| Elective admission | 0.944 | 0.126 | 0.664 | 0.727 | 1.226 |
| Year | 1.185 | 0.016 | <0.001 | 1.154 | 1.216 |
