# Supplementary material for: Trends and impact of antimicrobial resistance on older inpatients with urinary tract infections (UTIs): A national retrospective observational study
Source: PLoS One. 2019 Oct 3;14(10):e0223409. doi: 10.1371/journal.pone.0223409 (PMC6776395; doi:10.1371/journal.pone.0223409)
Supplement: S3 Appendix — (DOCX) [file pone.0223409.s003.docx]

**S3 APPENDIX. SENSITIVITY ANALYSES**


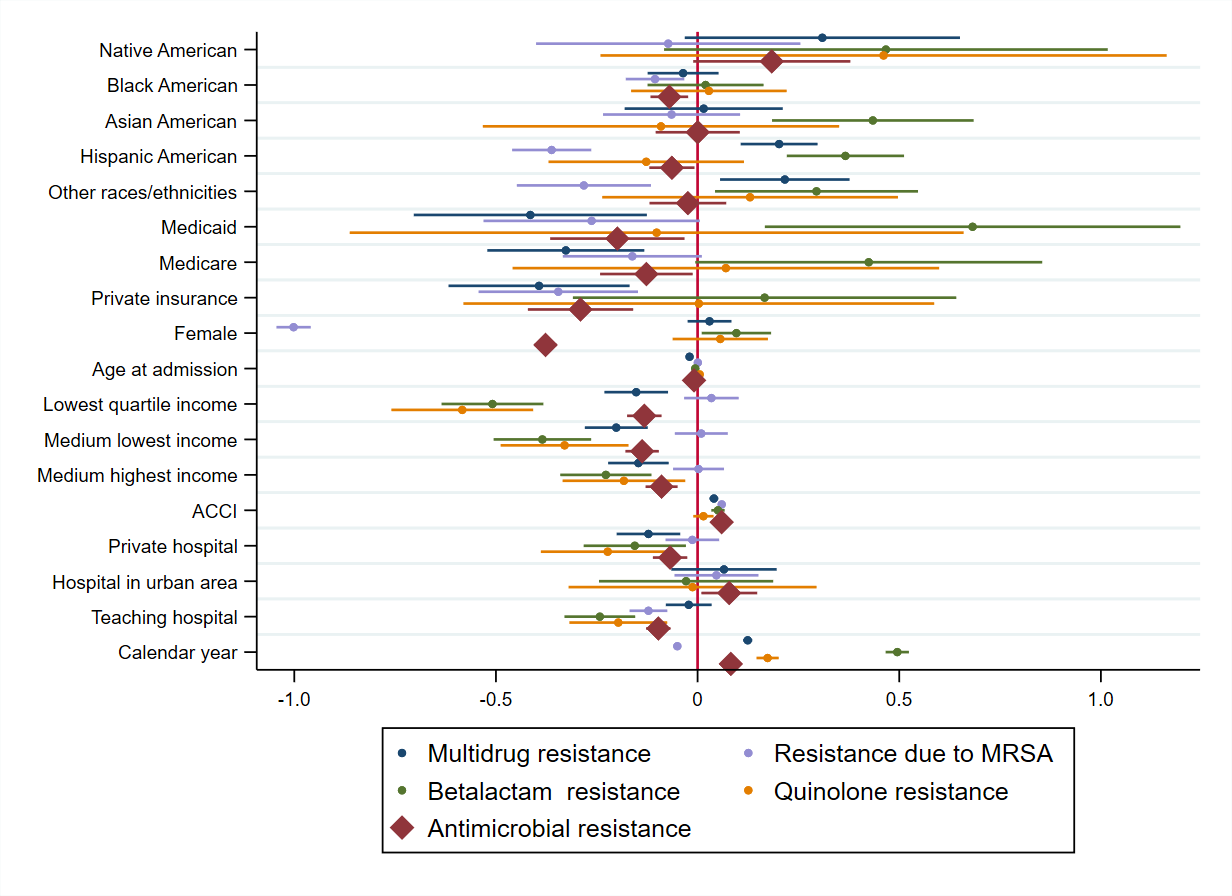


**Figure S1.** Factors associated with AR, BR, resistance due to MRSA, MR and QR in the sensitivity analyses.

Note: AR: antimicrobial resistance, BR: beta-lactam resistance, MRSA: Methicillin-resistant *Staphylococcus aureus*, MR: multidrug resistance, QR: quinolone resistance. AR group includes those with BR, resistance due to MRSA, MR, and QR.

**Table S2.** Impact of antimicrobial resistance

|  | **AR** | **BR** | **MRSA** | **MR** | **QR** |
| --- | --- | --- | --- | --- | --- |
| **Unadjusted models** | | | | | |
| All-cause inpatient mortality^a^, OR (95%CI) | 1.28  (1.15-1.43) | 0.52  (0.31-0.86) | 2.05  (1.79-2.34) | 0.69  (0.52-0.91) | 0.85  (0.49-1.46) |
| Discharge to healthcare facilities^a^, OR (95%CI) | 1.74  (1.69-1.79) | 1.43  (1.31-1.56) | 2.17  (2.07-2.28) | 1.4  (1.32-1.47) | 1.19  (1.07-1.34) |
| Length of stay^b^  Days (95%CI) | 1.74  (1.67 to 1.82) | 0.97  (0.64 to 1.29) | 2.36  (2.23 to 2.49) | 1.22  (1.11 to 1.33) | -0.12  (-0.28 to 0.04) |
| Hospital costs^c^  2016 USD (95%CI) | 2673  (2535 to 2811) | 1621  (1303 to 1939) | 3854  (3601 to 4106) | 1496  (1274 to 1717) | 76  (-226 to 379) |
| **Adjusted models^Φ^** | | | | | |
| All-cause inpatient mortality^a^, OR (95%CI) | 1.01  (0.9-1.14) | 0.5  (0.3-0.85) | 1.29  (1.12-1.49) | 0.67  (0.5-0.9) | 0.82  (0.46-1.46) |
| Discharge to healthcare facilities^a^, OR (95%CI) | 1.74  (1.69-1.8) | 1.41  (1.28-1.54) | 2.12  (2.01-2.24) | 1.48  (1.4-1.57) | 1.2  (1.06-1.36) |
| Length of stay^b^  Days (95%CI) | 1.11  (1.05 to 1.18) | 0.76  (0.45 to 1.08) | 1.35  (1.25 to 1.44) | 0.81  (0.72 to 0.9) | -0.06  (-0.22 to 0.1) |
| Hospital costs^c^  2016 USD (95%CI) | 1236  (1152 to 1320) | 911  (667 to 1156) | 1609  (1474 to 1745) | 611  (471 to 751) | 135  (-113 to 383) |
| *Note: ^a^ Logistic models; ^b^ Negative binomial regression model, ^c^ Generalized linear models, ^Φ^Models were adjusted for a range of socio-demographic and clinical covariates as stated in the Method section. All models were weighted for HCUP weights to generate national estimates, the reference cases were those without antimicrobial resistance. OR: odds ratio, 95%CI: 95% confidence interval. AR: antimicrobial resistance, BR: beta-lactam resistance, MRSA: resistance due to MRSA, MR: multidrug resistance, QR: quinolone resistance. AR group includes those with BR, resistance due to MRSA, MR, and QR.* | | | | | |
